# Disseminated Tuberculosis Resulting in Septic Shock in an Immunocompetent Patient

**DOI:** 10.7759/cureus.28025

**Published:** 2022-08-15

**Authors:** Marjorie F Jaffet, Mustapha Abubakar, Yakub Ibrahim, Udoka Ogbuneke, Wint Wahoo

**Affiliations:** 1 General Medicine, Mid and South Essex University Hospital NHS Trust, Southend-on-Sea, GBR; 2 Accident and Emergency, Mid and South Essex University Hospital NHS Trust, Southend-on-Sea, GBR

**Keywords:** scrofula, immunocompetent adult, disseminated tuberculosis, septic shock, septic shock in disseminated tuberculosis

## Abstract

Tuberculous infection (TB) is rare in the United Kingdom (UK) with a prevalence rate of 7.3 per 100,000 population in 2020 according to Public Health England. Tuberculous infection of any kind is more common in individuals born in TB-endemic areas. This report describes the case of a male with no significant past medical history who presented with shortness of breath and supraclavicular lymphadenopathy and was subsequently diagnosed with culture-positive disseminated TB. He developed septic shock, underwent treatment and improved. This case highlights an atypical patient profile for the diagnosis of disseminated TB with septic shock and draws attention to the challenges of diagnosing tuberculosis in TB-non-endemic areas. Clinicians should have a high index of suspicion for disseminated tuberculous infection in patients with chronic symptoms and signs affecting multiple organ systems without any obvious cause.

## Introduction

Disseminated tuberculosis (TB) can affect almost any part of the body with the commonest extrapulmonary site involved being the lymph node [1]. Some of the risk factors associated with disseminated TB include HIV infection, immunosuppression, Black ethnicity, female sex and living in TB-endemic areas [2]. A tuberculosis infection affecting two or more non-contiguous sites resulting from hematogenous or lymphatic spread is described as disseminated TB. Tuberculosis is very rare in the United Kingdom, with a rate of 7.3 per 100,000 population as recorded by Public Health England for 2020. Septic shock resulting from tuberculosis is rare, most reported cases have been noted in patients with low immunity [3]. Here we present a case of an immunocompetent man who was otherwise in good health with no significant risk factors for disseminated TB. He was subsequently diagnosed with disseminated TB after initial deterioration.

## Case presentation

A 69-year-old male presented with a history of progressive shortness of breath for one week. He also reported subjective fever, lethargy and generally feeling unwell for the same period of time. He noted a loss of weight over the 1-week period which he attributed to his reduced appetite. A review of other systems was unremarkable. His medical history included hypertension and benign prostatic hyperplasia. He is an ex-smoker with a 30-pack-year history, he last smoked approximately 27 years prior to this presentation. He had lived in Singapore in the late 1940s for 5 years and had been living in the United Kingdom ever since. He had received Bacillus Calmette Guerin (BCG) vaccine as a child. He reports no previous TB infection and no known history of recent travel to TB-endemic regions or contact with any persons with a chronic cough or TB disease. He was never incarcerated. He had a clinical frailty score of 1.

On examination, he was afebrile with a temperature of 36.9 degrees Celsius with a left non-tender, fixed, firm supraclavicular lymphadenopathy measuring 3 cm in diameter. His oxygen saturation on air was 99% and he was not tachypnoeic but had bilateral lower lung zone coarse crepitations. Other systemic examinations were unremarkable. On initial admission, the full blood count reveals haemoglobin of 90 g/L, C reactive protein (CRP) of 50 mg/L, with White cell count (WBC) and neutrophil count of 12 x 109/L and 9 x 109/L respectively. The initial chest radiograph was unremarkable.

**Figure 1:**
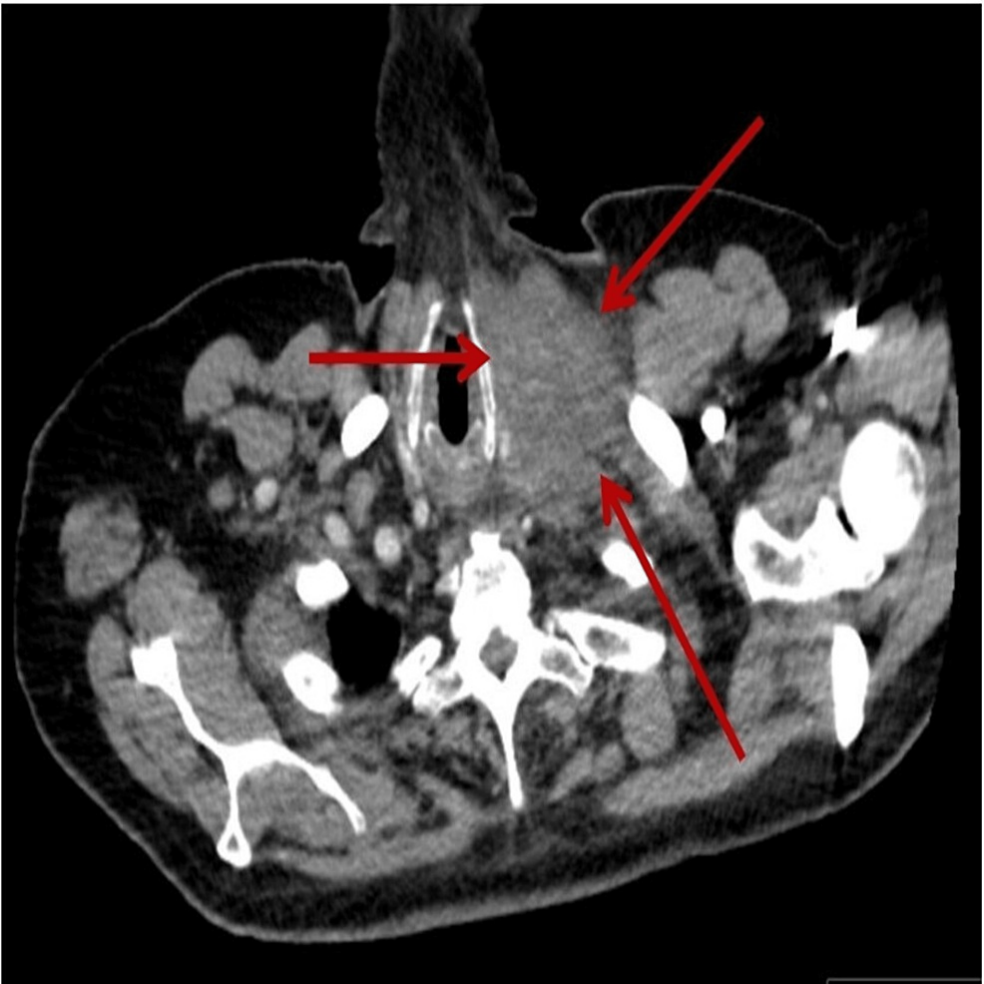
CT chest axial view showing a large ill-defined left supraclavicular mass (area pointed at by the three arrows)

**Figure 2:**
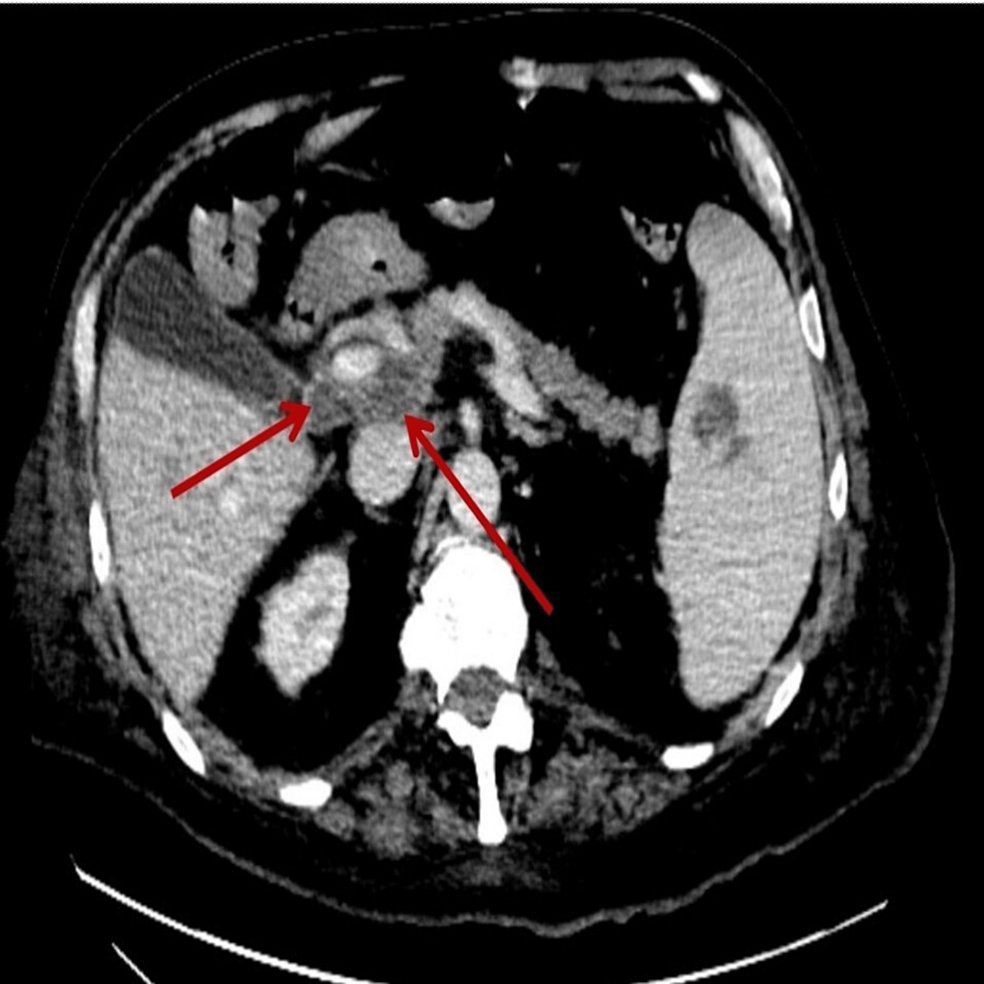
CT abdomen axial view revealing a large portocaval lymph node (arrows) and low attenuation suggesting a large necrotic node with splenic lesion

**Figure 3:**
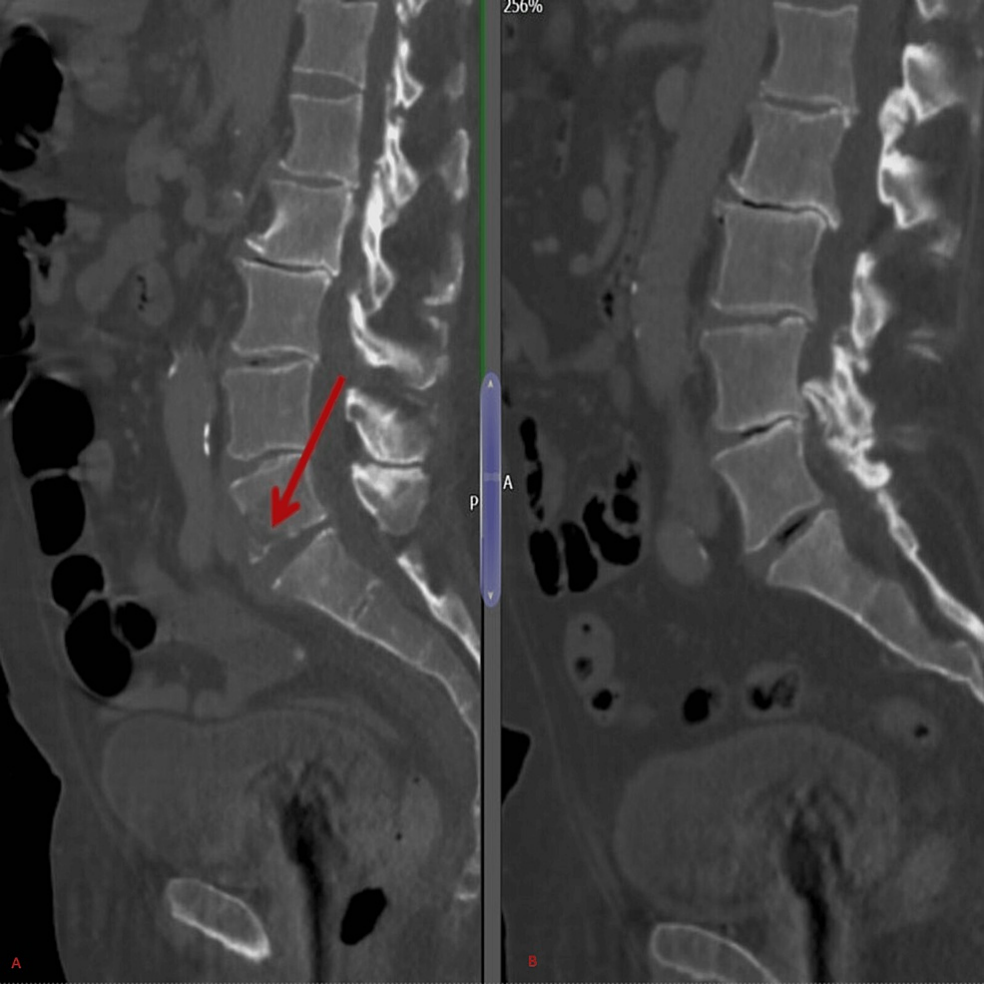
CT abdomen sagittal view showing an Ill-defined bone lesion (arrow) in L5 vertebral body (A) compared to a previous scan the patient had few months prior to admission (B)

While being treated on admission for bacterial pneumonia to rule out malignancy (giving the lymph node finding), a Well's score of 4.5 necessitated a Computer tomography (CT) pulmonary angiogram which ruled out pulmonary embolism but revealed a left-sided supraclavicular mass with subsequent CT abdomen and pelvis to investigate for malignancy showing lesions in the liver, spleen and vertebrae (Figures 1-3).

However, he improved clinically after a 5-day stay after admission and treatment with amoxicillin-clavulanate. The patient consented to have the lymph node biopsy done to investigate for cancer only after he recovered from his acute illness. However, on the ninth day, he deteriorated with worsening shortness of breath, confusion, increasing fever with the left supraclavicular lymphadenopathy showing signs of ulceration. On examination, he was febrile at 38 degrees Celsius, clinically pale, tachypnoeic, and tachycardic, with a saturation of 78% on air requiring supplemental oxygen, with stable blood pressure. He was treated as a case of sepsis with the ulcerated supraclavicular mass as the focus of infection. He deteriorated with a Glasgow coma score of 14/15 still tachypnoeic and tachycardic with hypotension refractory to fluid resuscitation requiring admission to the high-dependency unit (HDU) with vasopressor support.

Repeat blood tests showed CRP of 183 mg/L, haemoglobin of 76 g/L, WBC and neutrophil count of 15.5 x109/L and 12.69 x 109/L, respectively. Repeat chest radiograph revealed bilateral pleural effusion. The culture of the patient's urine, blood and sputum (including acid and alcohol fast bacilli) detected no growth. He was commenced on piperacillin-tazobactam, which was continued in the HDU as advised by the in-hospital microbiologist, together with metaraminol as vasopressor and mechanical ventilation.

A biopsy of the left supraclavicular lymph node revealed features in keeping with an abscess with granulation tissue and granulomatous response with no evidence of malignancy. He was counselled for a retroviral disease test which came back negative.

He was started on rifampicin, isoniazid, ethambutol and pyrazinamide with adjunctive oral prednisolone and pyridoxine to prevent isoniazid-induced neuropathy. He improved clinically requiring no organ support and was stepped down to the ward to continue his antitubercular medication. He spent additional 3 weeks in the ward (with isolation) prior to discharge to continue anti-tuberculosis medication.

## Discussion

This case highlights the challenges of diagnosing tuberculous infections, particularly in non-endemic regions and the need to initiate appropriate treatment in a timely fashion to improve patients’ chances of survival. The diagnosis was delayed as we initially thought the left-sided supraclavicular mass or lymph node enlargement is likely to be arising from a malignancy rather than from a tuberculous infection, more so in regions with less incidence and prevalence of tuberculous infection such as the UK. The lymph node biopsy was scheduled to be done shortly after his initial acute illness while still on admission and prior to his deterioration requiring HDU care. With the ulceration of the lymph node and worsening condition despite antibiotics, a clinical decision was made in the patient's best interest and with his next of kin informed to perform the biopsy as part of the investigation for infection and also malignancy.

In countries with low incidence and prevalence of mycobacteria tuberculosis infection, diagnosis is often delayed with increased morbidity and mortality [4-5]. As in our patient, it is often easily misconstrued with other common medical conditions, particularly in non-endemic areas [6].

Initially, it will present with symptoms and signs of the affected body system or with constitutional symptoms of low-grade fever, weight loss and lethargy. Tuberculous infection commonly presents with features of pulmonary involvement with the presence of shortness of breath and cough lasting for weeks. It can affect any part of the body with the lymphatic system involvement noted as the commonest extrapulmonary site involved [1]. Other sites include lungs, skin, bones, gastrointestinal system and the central nervous system. The presence of chronic unexplained symptoms not amenable to ongoing therapy should raise the suspicion of tuberculosis. Having lived in a tuberculosis-endemic region for a period of 5 years is a risk factor for tuberculosis, which has been known to stay dormant for decades, with reactivation occurring when the conditions are favourable. Bacillus Calmette Guerin (BCG) vaccine has been known to have a 50% effectiveness in the prevention of tuberculosis [7-8].

As in our patient, blood investigation would often reveal features of anaemia (often of chronic illness) with low haematocrit and raised C reactive protein or erythrocyte sedimentation rate. Chest radiograph may be unremarkable or may reveal features of hilar adenopathy, pleural effusion together with cavitary lesions. Though our patient had no features of being immunocompromised, a test for acid or alcohol fast bacilli may return as negative, particularly in patients who are immunocompromised [9].

The presence of isolated painless lymph node enlargement lasting for more than 3 to 4 weeks even without clinical features suggestive of malignancy warrants further investigation [10]. In our patient, CT abdomen and pelvis showed involvement of the liver and spleen while excisional biopsy of the supraclavicular lymph node revealed caseating granuloma.

Extrapulmonary tuberculosis has been noted as an independent risk factor for increased morbidity, and If untreated, may result in sepsis necessitating intensive care [11]. Thus, a high index of suspicion together with timely treatment of disseminated mycobacterium infection is essential to reduce mortality and morbidity [12].

## Conclusions

Disseminated tuberculosis can present a diagnostic challenge particularly in non endemic regions, while disseminated tuberculosis resulting in sepsis and septic shock is rare, it been documented in the literature particularly among immunocompromised patients. Immunocompetent patients are also at risk of sepsis from undiagnosed and untreated disseminated tuberculosis with risk of morbidity and mortality as shown in this case.
